# *Porphyromonas gingivalis* short fimbriae are regulated by a FimS/FimR two-component system

**DOI:** 10.1111/j.1574-6968.2007.00722.x

**Published:** 2007-04-20

**Authors:** Jie Wu, Xinghua Lin, Hua Xie

**Affiliations:** School of Dentistry, Meharry Medical College Nashville, TN, USA

**Keywords:** gene regulation, *Porphyromonas gingivalis*, two-component system

## Abstract

*Porphyromonas gingivalis* possesses two distinct fimbriae. The long (FimA) fimbriae have been extensively studied. Expression of the *fimA* gene is tightly controlled by a two-component system (FimS/FimR) through a cascade regulation. The short (Mfa1) fimbriae are less understood. The authors have recently demonstrated that both fimbriae are required for formation of *P. gingivalis* biofilms. Here, the novel finding that FimR, a member of the two-component regulatory system, is a transcriptional activator of the *mfa1* gene is promoted. Unlike the regulatory mechanism of FimA by FimR, this regulation of the *mfa1* gene is accomplished by FimR directly binding to the promoter region of *mfa1*.

## Introduction

*Porphyromonas gingivalis* is a gram-negative bacterium, which is considered to be a major periodontal pathogen (Socransky & Haffajee, 2005). It is also a pathogen that may be involved in coronary heart disease and preterm births (Boggess *et al.*, 2005; Brodala *et al.*, 2005; Chou *et al.*, 2005). The ability of *P. gingivalis* to initiate a periodontal infection is mainly dependent on the expression of fimbriae (Malek *et al.*, 1994). Two distinct fimbriae are found on the surface of the organism (Dickinson *et al.*, 1988; Hamada *et al.*, 1996). The long (major) filamentous structure is comprised of a FimA subunit protein encoded by the *fimA* gene. The short (minor) fimbriae are made up of a 67 kDa protein (Mfa1). Both fimbriae appear to be involved in bacterial pathogenicity (Amano *et al.*, 2004).

The function of the FimA protein and regulation of *fimA* expression have been extensively studied. The FimA protein is required for *P. gingivalis* colonization on salivary coated surfaces, and the early colonization of dental plaque (Malek *et al.*, 1994; Levesque *et al.*, 2003; Maeda *et al.*, 2004). A *P. gingivalis fimA* mutant shows impaired invasion capability of epithelial cells compared with wild-type strain, suggesting the involvement of FimA in the bacterial interaction with surface receptor(s) on gingival cells (Weinberg *et al.*, 1997). Earlier studies by the authors showed that FimA expression was modulated by environmental cues, including temperature and hemin concentration, and by the presence of *Streptococcus cristatus*, an early colonizer of dental plaque (Xie *et al.*, 1997, 2000b). FimR, a response regulator of the *fimS/fimR* two-component system was identified, and FimA expression was found to be dramatically reduced in *fimR* mutants (Hayashi *et al.*, 2000). Investigation of the mechanism of regulation of *fimA* by FimR indicates that FimR does not bind directly to the *fimA* promoter, but rather binds to the promoter region of the first gene (*pg2130*) in the *fimA* cluster, suggesting that PG2130 is the FimR target gene, which in turn regulates expression of other genes in the *fimA* cluster, including the *fimA* gene (Nishikawa *et al.*, 2004).

The short fimbriae (Mfa1) also contribute to *P. gingivalis* colonization. Coadhesion and biofilm development between *P. gingivalis* and *Streptococcus gordonii* require the interaction of Mfa1 with streptococcal protein SspB (Park *et al.*, 2005). The authors have recently reported that the short fimbriae are required for *P. gingivalis* cell–cell aggregation, an essential step in microcolony formation (Lin *et al.*, 2006). A mutant with a deficiency in minor fimbriae binds to a saliva-coated surface but does not form microcolonies as the wild-type strain does. Mfa1 expression appears to fluctuate under various growth conditions (Masuda *et al.*, 2006). In a nutrient-limited medium, expression of FimA and Mfa1 are inhibited in *P. gingivalis*, whereas such differences are not found in gingipain expression. A recent study has shown that expression of *mfa1* is repressed in the presence of some common oral plaque bacteria such as *S. gordonii*, *Streptococcus sanguinis* and *Streptococcus mitis* (Park *et al.*, 2006). However, very little is known about regulatory mechanisms of *mfa1* expression.

In this study, it is demonstrated that FimR is a positive regulator of Mfa1 expression. Evidence is provided that unlike FimR-dependent *fimA* expression, FimR regulates *mfa1* expression by directly binding to the promoter region of *mfa1*.

## Materials and methods

### Bacterial strains and growth conditions

The bacterial strains and plasmids used in this study are listed in Table 1. *Porphyromonas gingivalis* 33 277 and its derivatives were grown from frozen stocks in trypticase soy broth (TSB) or on TSB blood agar plates, supplemented with yeast extract (1 mg mL^−1^), hemin (5 μg mL^−1^) and menadione (1 μg mL^−1^), at 37°C in an anaerobic chamber (85% N_2_, 10% H_2_, 5% CO_2_). *Escherichia coli* DH5α was the host for plasmids, and grown in Luria–Bertani (LB) broth at 37°C. Antibiotics were used, when appropriate, at the following concentrations: gentamicin (100 μg mL^−1^), erythromycin (5 μg mL^−1^), ampicillin (50 μg mL^−1^), kanamycin (50 μg mL^−1^) and tetracycline (10 μg mL^−1^).

**Table 1:** Strains and plasmids used in this study

Strains and plasmid	Relevant characteristics*	Source or reference
Strains		
*P. gingivalis*		
33277	Type strain from ATCC	Lab collection
FAT	*P. gingivalis* mutant with the *fimA* gene inactivated by insertion – the tetracycline the *tetA*(*Q*)gene, Tet^r^	Lin *et al*. (2006)
MFAE	*P. gingivalis* mutant with the *mfa1* gene inactivated by insertion – a *ermF*–*ermAM* cassette, Em^r^	Lin *et al*. (2006)
FRE	*P. gingivalis* mutant with the *fimR* gene inactivated by insertion – a *ermF*–*ermAM* cassette, Em^r^	This study
*E. coli*		
DH5α	F^−^ ϕ80d*lac*ZΔ(*lacZYA–argF*)U169 *endA1* *sup*E44 *recA1* *relA1*	BRL
Plasmids		Lee *et al*. (1996)
pVA3000	Suicide vector containing an *ermF*–*ermAM* cassette	Lee *et al*. (1996)
pBSK1.2-5	pUC19 containing a *tetA*(Q)2 gene	Lepine *et al*. (1996)
PCRII-TOPO	Linearized plasmid with single 3′ dT residues, Km^r^ Am^r^	Invitrogen
pFR	PCRII-TOPO plasmid carrying a *fimR* gene	This study
pFRE	pFR plasmid an *ermF*–*ermAM* cassette inserted in the *fimR* gene	This study

* Km^r^, Tet^r^, Em^r^, Am^r^, resistance to kanamycin, tetracyline, erythromycin, ampicillin.

### Construction of the *fimR***^−^** mutant

An insertional *fimR* mutant was constructed by allelic replacement. Briefly, the *fimR* gene was amplified by PCR using primers fimRF and fimRR (Table 2) and cloned into pCRII-TOPO (Invitrogen, Carlsbad, CA) to give rise to a pFR plasmid (Table 1). A 2.1-kb *ermF*–*ermAM* cassette (Fletcher *et al.*, 1995) was amplified using plasmid pVA3000 as a template and ErmF and ErmR as primers, which introduced NdeI sites at both ends of the PCR product. The *ermF*–*ermAM* cassette was then inserted into the *fimR* gene cloned in plasmid pFR. The resulting plasmids pFRE were linearized with XhoI and introduced into *P. gingivalis* 33277-by electroporation. Electroporation was carried out by a modification of the procedure of Fletcher *et al.* (1995). *Porphyromonas gingivalis* 33277 competent cells were obtained by suspending early-log-phase cells in electroporation buffer (10% glycerol, 1.0 mM MgCl_2_). The cells were incubated with the linearized plasmid and were pulsed with a Bio-Rad gene pulser (Hercules, CA) at 2.5 kV. The cells were then immediately added to the TSB, and incubated anaerobically for 16 h. The *fimR***^−^** mutants (FRE) resulting from a double-cross-over recombination were selected on Trypticase soy agar plates containing erythromycin (5 μg mL^−1^). The insertional mutation was confirmed by PCR analysis.

**Table 2:** Oligonucleotide primers

Gene	Primer name	Primer sequences (5′–3′)	Application
*fimR*	rfimR-F	ATGATTAGTATCGTACTC	The full-length *fimR* ORF amplification
	rfimR-R	CTATTGCCAATCCACTAA	
	fimRF	TAGGCTTTTGCCAGATTGGA	Construction of *fimR* mutation
	fimRR	CCAAATCGGGAATTTAGCTC	
*ermF-ermAM*	ErmF	CACCGTCATATGCGATAGCTTCCGCTATTGCT	*ermF-ermAM* cassette amplification
	ErmR	GGAACTCATATGTCCCCGAAGCTGTCAGTAGT	
*fimA*	fimAF	CGGAACGAATAACCCAGAGA	Real-time PCR
	fimAR	CTGACCAACGAGAACCCACT	
	fimAProm-F	CGACGCTATATGCAAGACAA	The biotin-labeled promoter region
	fimAProm-R	Bio-TGTAACGGGTTCTGCCTCGT	
*Mfa1*	MfaProm-F	CTCTCGCGAGGGTCAATATC	The biotin-labeled promoter region
	MfaProm-R	Bio-CGTCTTACCGGCTTCCCTAT	
	67KD121F	CAGATGGGTTGTTGCTCA	The biotin-labeled coding region
	67KD121R	Bio-ATAGAAAGTGCTGCTGGTAG	
	MfaF	CAGATGGGTTGTTGCTCA	Real-time PCR
	MfaR	GAAAGTGCTGCTGGTAG	
	MfaTSR1	CTCGTTATCACATATCCGAACC	Identification of transcriptional start site
	MfaTSR2	GAAGCAAAGCCCAATGAGAG	
	MfaTSR3	CCGCTCGACTCACGAGACTA	
	MfaTSR4	CACGACATAGAGTGTTCAGA	
	MfaTSR5	CGTCTTGCCGACAGCAGAAT	
	MfaTSF1	AGCCGGTAAGACGTAGCTGA	
	MfaTSF2	ACGTAGAAGACAGCAGAATA	
	MfaTSF3	TCTCTCGCGAGGGTCAATA	
*16S*	16sRNA-F	TGGGTTTAAAGGGTGCGTAG	Real-time PCR
	16sRNA-R	CAATCGGAGTTCCTCGTGAT	
*rgpA*	rgpAF	CAACCAGTCTTGGGCTTCTC	Real-time PCR
	rgpAR	CCACCATAGCAAACATACCG	

### RNA isolation and qPCR

*Porphyromonas gingivalis* strains were grown anaerobically on Trypticase soy agar plates at 37°C for 48 h. *Porphyromonas gingivalis* cells were collected and mixed in Trizol Reagent (Invitrogen). The RNA in the supernatant was then purified using an RNeasy mini spin column (Qiagen, Valencia, CA). To minimize contamination with genomic DNA, RNA samples were digested on the column with RNase-free DNase. The total RNA was tested using an Agilent 2100 Bioanalyzer to insure the quality of the samples. Gene expression was measured using the QuantiTect SYBR Green reverse transcriptase polymerase chain reaction (RT-PCR) Kit (Qiagen) and the iCycler iQ real-time detection system (Bio-Rad Laboratories, Inc.) according to the manufacturer's instructions. The primers for the *fimA* gene were fimAF and fimAR, and for the *mfa1* gene were mfa1F and mfa1R. Expression of the 16S rRNA gene and *rgpA* (a gene encoding for arginine-gingipain) were tested as a control to normalize samples for variations in sample volume loading. Amplification reactions consisted of a reverse transcription cycle at 50°C for 30 min, an initial activation at 95°C for 10 min, and 40 cycles of 94°C for 15 s, 60°C for 30 s and 72°C for 30 s. For quantitative analysis of gene expression, fivefold serial dilutions of total RNA, from 0.4 to 250 ng, were used as the template in each 50-μL reaction to generate a standard curve. Data were collected only from the reactions in which correlation coefficients of standard curves were ≥0.99 and where the melting curves showed a single peak. Values represent the mean±SD of duplicate samples obtained from three independent experiments.

### Western blot analysis

*Porphyromonas gingivalis* strains were grown on TSB blood agar plates for 48 h. The surface proteins were collected by sonication and centrifugation as described previously (Xie *et al.*, 2004). Protein concentrations of the samples were determined using a Bio-Rad protein assay. The soluble proteins (5 μg) were separated by 12% sodium dodecyl sulfate-polyacrylamide gel electrophoresis (SDS-PAGE), along with prestained MW standards (Bio-Rad) and were transferred to nitrocellulose membranes (Gibco BRL) with Mini Transblot Electrophoretic transfer cell (Bio-Rad Laboratories) at 100 V for 1 h. The membrane was treated with 30 mL of blocking solution [3% bovine serum albumin (BSA) in phosphate-buffered saline (PBS) containing 0.1% Tween-20, pH 7.4] for 1 h and incubated for 1 h with a polyclonal anti-FimA or anti-Mfa1 antibody diluted 1 : 1000 in PBS containing 0.1% Tween-20, pH 7.4. The membrane was then rinsed twice and washed three times for 15 min each with 0.1% Tween-20 in PBS. The membrane was incubated with antirabbit horseradish peroxidase-conjugated secondary antibodies for 1 h and rinsed and washed as described above. Antigen–antibody reactivity was visualized by enhanced chemiluminescence (GE Healthcare Bio-Sciences Corp, Piscataway, NJ).

### 5′ RACE analysis of Mfa1 transcripts

The transcriptional start site of *mfa1* was determined using a FirstChoice RLM-RACE Kit (Ambion, Austin, TX). Briefly, a 45 base 5′ RNA adapter oligonucleotide was ligated to the 5′ end of the total RNA (10 μg) using T4 RNA ligase. Reverse transcription (RT) of cDNA was performed using M-MLV reverse transcriptase with primer MfaTSR2 of *mfa1*. Nested PCR was performed by first using 5′ RACE outer primer and *mfa1*-specific reverse primer MfaTSR2 to amplify 5′ adapter-linked cDNA molecules of *mfa1*. Inner PCR was then conducted with 5′ RACE inner primer and MfaTSR1, and with the PCR product generated from the outer primers as templates. Five microliters of each PCR reaction was analyzed by 1.5% agarose gel electrophoresis. The PCR fragments of the inner PCR product were extracted and cloned into a pCRII-TOPO vector (Invitrogen) and sequenced using ABI capillary sequencer (Perkin-Elmer).

### FimR cloning and expression in *E. coli*

DNA fragments of the *fimR* were amplified by PCR with primers 5′-ATGATTAGTATCGTACTC and 5′-CTATTGCCAATCCACTAA, which produced a 684 bp PCR product. The PCR products were then cloned into pCRII-TOPO. Recombinant FimR (rFimR) was expressed in *E. coli* using a pET protein expression system (Novagen, San Diego, CA). The FimR DNA fragment was subcloned into the pET-30b down stream of a histidine tag. The recombinant FimR was expressed in *E. coli* BL21 (DE3) cells carrying the pET-30b/FimR plasmid in the presence of IPTG and kanamycin. His-tagged rFimR was purified with Ni^2+^-charged His-bind resin. The His-tag on the recombinant protein was cleaved with enterokinase and removed by His-bind resin. Enterokinase was then removed using Ekapture agarose.

### Electrophoretic mobility shift assay (EMSA)

EMSAs were performed using the LightShift Chemiluminescent EMSA Kit (PIERCE, Rockford, IL) according to the manufacturer's instructions. Biotin-labeled DNA fragments were generated using 5′ biotin-incorporated primers (Invitrogen). For phosphorylation, rfimR was incubated with binding buffer containing 50 mM acetylphosphate lithium potassium salt (Sigma, Saint Louis, MO) at room temperature for 30 min. Binding of rFimR to DNA was carried out in a 20-μL reaction mixture containing 20 fmol biotin-labeled DNA, 10 mM Tris, pH 7.5, 50 mM KCl, 1 mM dithiothreitol, 10 ng μL^−1^ poly (dI–dC), 2% glycerol, 0.05% NP-40, 2 mM MgCl with various amounts of purified rFimR protein (10, 20 and 40 pmol μL^−1^) at room temperature for 30 min. Samples were then loaded and run into a 5% nondenaturing polyacrylamide gel in 0.5 × TBE buffer. The electrophoresis was carried out for 2 h at a constant 100 V at 4°C. The DNA and protein complexes were then transferred to a positively charged nylon membrane (380 mA, 30 min). The biotin end-labeled DNA was detected using the streptavidin–horseradish peroxidase conjugate and the chemiluminescent substrate. Each EMSA experiment was repeated at least three times.

## Results

### Role of FimR in *mfa1* expression

**Fig. 1:**
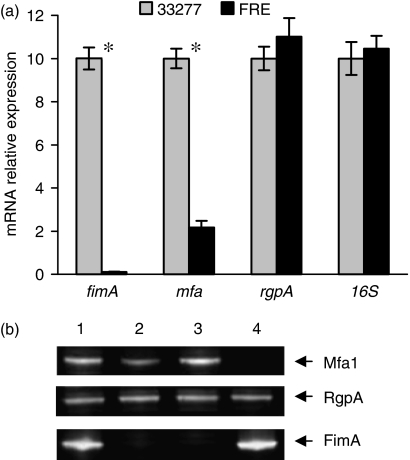
Expression of fimbrial genes in *Porphyromonas gingivalis*. (a) Fold changes in gene transcription in wild type strain 33277 and the *fimR***^−^** mutant were measured by real-time PCR. Primers used for each gene are shown in Table 2. Results shown are means and SDs from triplicate experiments. Fold differences were calculated using the relative comparison method. Significant differences (*P*<0.001 by *t* test) are labeled with asterisks. (b) Western blot analyses show the expression of Mfa1, RgpA, and FimA in wild type 33 277 (lane 1), the *fimR***^−^** mutant FRE (lane 2), the *fimA***^−^** mutant FAT (lane 3), and the *mfa1***^−^** mutant MFAE (lane 4).

The *fimA* gene is the only gene known to be tightly controlled by the FimS/FimR system. It was postulated that the expression of other genes may also be controlled by this two component regulatory system. To investigate effects of FimR on expression of the *mfa1* gene, an insertional *fimR* mutant was constructed by allelic replacement. Expression of *fimA* and *mfa1* in the *fimR***^−^** mutant was determined using real-time PCR analysis. Statistically significant differences of the level of gene expression in 33277 and the *fimR***^−^** mutant were calculated by a Student's *t*-test. As shown in Fig. 1a, expression of the *fimA* gene was abolished in the *fimR***^−^** mutant strain FRE. This result is consistent with previous observations (Hayashi *et al.*, 2000; Nishikawa *et al.*, 2004). The striking finding is that expression of the *mfa1* gene was also repressed threefold in the *fimR***^−^** mutant, although not to the degree observed with the *fimA* expression. However, the *fimR***^−^** mutation had no effect on expression of *rgpA*, a gene encoding the arginine-specific protease, or the *P. gingivalis* 16S RNA gene. This analysis suggests the FimS/FimR system is required for expression of both major and minor fimbriae.

To determine production of long (major) and short (minor) fimbriae in the *fimR***^−^** mutant, western blotting was performed with a polyclonal anti-FimA or anti-Mfa1 antibody to compare fimbrial production in wild-type strain (33277), the *fimR***^−^** mutant (FRE), the *fimA***^−^** mutant (FAT) and the *mfa1***^−^** mutant (MFAE). Density of protein bands was determined by UVP Bioimaging System (UVP, CA). This analysis revealed that the expression of the *fimA* and *mfa1* genes was consistent at the mRNA level and protein level (Fig. 1b). FimA protein was not detectable in the *fimR***^−^** mutant, while a 50% lower level of Mfa1 protein was found in the *fimR***^−^** mutant compared with that in wild-type strain 33277. Similarly, there was no apparent change in RgpA production in the *fimR***^−^** mutant, which was detected by anti-RgpA serum.

### Identification of the transcriptional start site of the *mfa1* gene

**Fig. 2:**
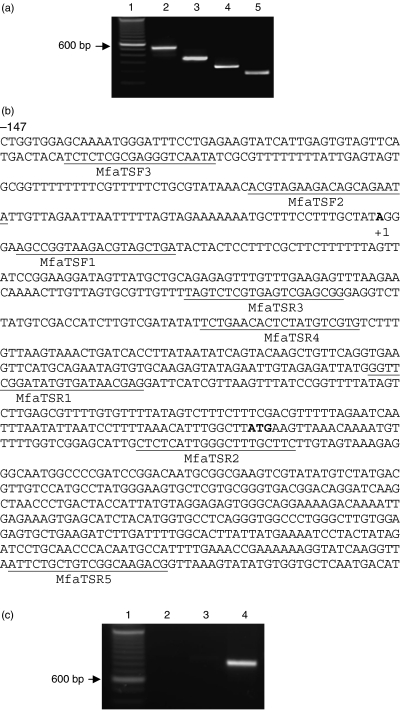
Determination of the transcriptional start site of *mfa1*. (a) Four different *mfa1* sense-strand primers (lanes 2–5, primers MfaTSR2, MfaTSR1, MfaTSR4, MfaTSR3, respectively) were used with 5′ RLM-RACE primers to determine the approximate transcriptional start for *mfa1*. The PCR products were visualized on a 1.5% agarose TAE gel containing ethidium bromide. Molecular weight standards (indicated in base pairs) are in lane 1. (b) DNA sequence of the *mfa1* promoter region. The transcriptional start site A (+1) and the potential start codon ATG are bolded. The primers used for RLM-RACE and RT-PCR are underlined. (c) RT-PCR analysis. Lane 1, 1 kb ladder marker; lane 2 is RT-PCR with MfaTSF3 (from −139 to −121) and MfaTSR5 (from +805 to +824); lane 3 is RT-PCR with MfaTSF2 (from −66 to −47) and MfaTSR5 (from +805 to +824); lane 4 is RT-PCR MfaTSF1 (from +6 to +25) and MfaTSR5 (from +805 to +824).

To identify the promoter region of *mfa1*, the transcriptional start site was first determined. The RACE experiment was first conducted with *mfa1*-specific reverse primers MfaTSR1 located at 135 bp up-stream of the potential start codon and MfaTSR2 located at 37 bp downstream of the potential start codon (Fig. 2a). The transcriptional start site (the A) of *mfa1* was at 434 bp upstream from the potential start codon (Fig. 2b). To verify the result, the RACE experiment was repeated with *mfa1*-specific reverse primers MfaTSR3 and MfaTSR4 located at 237 bp upstream of the potential start codon. The same transcriptional start site was identified.

To confirm the result of RACE, reverse-transcriptional PCR using three sets of primers was performed. As shown in Fig. 2c, the PCR product was generated only with primers MfaTSF1 (corresponding to +6 to +25) and MfaTSR5 (+805 to +824). There was no PCR product generated from RT-PCR using the primers (MfaTSF3, from −139 to −121 or MfaTSF2, from −66 to −47) which correspond to the DNA sequences upstream from the transcriptional start site. This transcriptional start site is 390 bp upstream of the site previously reported (Park *et al.*, 2006). It is likely that *mfa1* gene possesses two functional promoters, which are also detected in the *fimA* gene of *P. gingivalis* (Xie & Lamont, 1999; Nishikawa *et al.*, 2004).

### Binding of FimR to the promoter region of *mfa1*

**Fig. 3:**
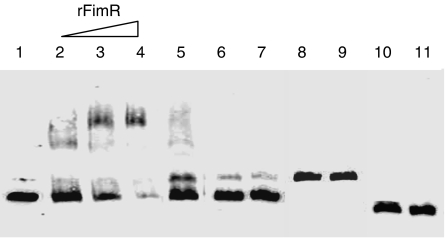
Nucleic acid binding properties of FimR. Electrophoretic mobility shift assays were performed using the biotin-labeled DNA probes. Lane 1, the biotin-labeled *mfa1* promoter region (156 bp) alone; lane 2–4, mobility of the biotin-labeled *mfa1* promoter region was noted in the presence of increasing amounts of rFimR protein (10, 20, and 40 pmol μL^−1^, respectively), as indicated on the top; lane 5, the biotin-labeled *mfa1* promoter region, rfimR 40 pmol μL^−1^, and 100-fold excess of unlabeled *mfa1* probe; lane 6 is the biotin-labeled *mfa1* promoter region alone; lane 7 is the biotin-labeled *mfa1* promoter region, and rHGP44 40 pmol μL^−1^; lane 8 is the biotin-labeled *fimA* probe (176 bp) alone; lane 9 is the biotin-labeled *fimA* probe (176 bp), and rFimR 40 pmol μL^−1^; lane 10 is the biotin-labeled *mfa1* coding region (121 bp) alone; lane 11 is the biotin-labeled *mfa1* coding region (121 bp) and rFimR 40 pmol μL^−1^.

The previous study has shown that the mechanism of FimR activation of the *fimA* gene involves a regulatory cascade (Nishikawa *et al.*, 2004). It was postulated that different mechanisms might be involved in FimR-mediated *mfa1* expression, since expression regulation of *mfa1* by FimR was not controlled as tightly as observed for *fimA* expression. One possibility is that FimR modulates *mfa1* expression by directly binding to the promoter region of *mfa1*. To test this hypothesis, electrophoretic mobility shift assays were performed. The *mfa1* promoter (positioned from +18 to −138), *fimA* promoter (positioned from −22 to−190) (Xie & Lamont, 1999) and *mfa1* coding DNA (positioned from +1253 to +1373) were generated by PCR with the 5′ biotin-labeled primers (Table 1). The recombinant FimR (rFimR) was expressed in pET expression system and purified from *E. coli*. The rHGP44 protein, a binding domain of *P. gingivalis* gingipains (Xie *et al.*, 2006), expressed in the same system and purified by the same procedures as rFimR was used as a control. Cold competitor chase experiments with a 100-fold excess of unlabeled DNA probe as a specific competitor were also used to demonstrate the specificity of rFimR binding. As shown in Fig. 3, the DNA fragment of the *mfa1* promoter region was shifted in the presence of the rFimR. Retarded *mfa1* promoter-rFimR complex was detected with as little as 10 pmol μL^−1^ rFimR (Fig. 3). As the concentration of rFimR increased, the retarded protein–DNA complex became evident, with complete loss of the *mfa1* promoter DNA. The unlabeled *mfa1* promoter fragments effectively competed with the labeled fragment, suggesting a specific interaction between rFimA and the *mfa1* promoter. To investigate the role of phosphorylation of FimR in its binding to the *mfa1* promoter region, EMSA experiments were also performed with the phosphorylated rFimR. No significant difference was detected in the level of DNA binding between the phosphorylated rFimR and unphosphorylated rFimR (data not shown). In agreement with a previous report (Nishikawa *et al.*, 2004), rFimR did not bind to the *fimA* promoter region, suggesting that regulation of *fimA* expression by FimR is through a different mechanism. Moreover, incubation of rHGP44 with *mfa1* promoter fragment did not retard the DNA movement in polyacrylamide gel. There was also no DNA shift detected when rFimR was incubated with the coding region of *mfa1*. These data clearly show that FimR protein can bind specifically to the *mfa1* promoter region, acting as an activator of *mfa1* transcription. EMSA experiments were also performed to examine whether the rFimR binds to the other promoter region identified by Park *et al.* (2006). The biotin-labeled DNA fragment corresponding to this promoter region did not shift in the presence of the rFimA protein (data not shown), suggesting that only the promoter identified here is involved for *mfa1* expression mediated by FimR.

## Discussion

The two-component regulatory system is a major mechanism of signal transduction and is widespread in bacteria. Six putative two-component regulatory systems were detected by surveying the *P. gingivalis* W83 genome database for homologues of the two-component sensor histidine kinase (Hasegawa *et al.*, 2003). Although most target genes of *P. gingivalis* two-component systems are unknown, the role of the FimS/FimR in expression of the *fimA* gene is well defined. Expression of minor fimbriae (*mfa1*) in a *fimR* mutanthas been investigated. A comparison of the transcriptional level of the *mfa1* in *P. gingivalis* wild-type strain and in the *fimR* mutant indicates that the FimS/FimR system is a positive regulator for the *mfa1* gene, although the system controls two fimbrial genes at different levels. It is hypothesized that the FimS/FimR system regulates expression of each fimbrial gene through a unique mechanism. The previous study suggested that regulation of *fimA* expression by FimR is through a regulation cascade involving interaction of FimR and the promoter region of the first gene in the *fimA* cluster (Nishikawa *et al.*, 2004). Here it is demonstrated that FimR binds directly to the promoter region of the *mfa1* gene, suggesting a direct role of FimR in activation of *mfa1* expression. It has also been reported previously that the transcriptional activity of *fimA* was reduced in the *fimA* mutant, indicating multiple levels of control of *fimA* expression in *P. gingivalis* (Xie *et al.*, 2000a). This may explain the much tighter control of *fimA* expression by FimR. However, the possibility cannot be excluded that other regulatory elements are also involved in expression of the *mfa1* gene.

A two-component regulatory system typically contains a membrane-bound histidine kinase sensor and a cytosolic response regulator. Phosphorylation, mediated by histidine kinase at a specific aspartate residue, activates DNA-binding activity of the response regulator and initiates the corresponding cellular response. However, no apparent difference was found in DNA-binding affinity between rFimRs with or without acetyl phosphate treatment. Observation suggests that different mechanisms may be involved in *P. gingivalis* FimR activation. Activation of a regulatory protein not corresponding to phosphorylation was also observed in *Streptococcus mutans* (Biswas & Biswas, 2006). Phosphorylation of CovR, a global response regulator, had no effect on its DNA-binding affinity. The fact that FimR was not activated by phosphorylation may also be due to the short lifetime of the phosphorylated state, which has been observed in other bacteria (Stock *et al.*, 2000).

The transcriptional start site of the *mfa1* gene located at 434 bp upstream of the putative start codon was detected, which is also 390 bp upstream of the site previously reported (Park *et al.*, 2006). The transcriptional site revealed here is confirmed by RT-PCR analysis. Data of this study suggest that transcription of the *mfa1* gene originated at a distal upstream transcriptional start site and read through the promoter region suggested by Park *et al.* (2006). Moreover, FimR appears to act on the promoter region identified here, suggesting that this promoter may make significant contributions toward *mfa1* expression through the FimS/FimR system. Gene expression under the control of two promoters is not uncommon in bacteria. In *E. coli*, two promoters direct transcription of *acs* encoding, an acetate-scavenging enzyme required for fitness during periods of carbon starvation – the distal *acs*P1 and the proximal *acs*P2 (Beatty *et al.*, 2003). It is suggested that each promoter may interact with different regulatory elements. Two promoter regions in the *P. gingivalis fimA* gene were also reported (Xie & Lamont, 1999; Nishikawa *et al.*, 2004). A cascade regulation starting with FimR appears to act on the upstream promoter (Nishikawa *et al.*, 2004). The observations that FimR binds only to the upstream promoter region of the *mfa1* gene and that activity of the downstream promoter is inhibited by *S. gordonii*, *S. sanguinis* and *S. mitis* (Park *et al.*, 2006) imply the complexity of regulation of *mfa1* expression. It is possible that two promoters of *mfa1* are regulated in response to different environmental signals. The hypothesis is currently under investigation.

In conclusion, *P. gingivalis* fimbriae play a predominant role in the attachment of the organism to a variety of oral surfaces (Lamont & Jenkinson, 2000; Amano *et al.*, 2004), although other surface proteins, such as gingipains, may also be involved in the bacterial colonization (Tokuda *et al.*, 1996; Chen *et al.*, 2001). It has been recently reported by the authors that both major fimbriae and minor fimbriae contribute to the formation of *P. gingivalis* biofilm (Lin *et al.*, 2006). Major fimbriae are required for initial attachment and the minor fimbriae appear to play an important role in microcolony formation by facilitating cell–cell interactions. The data presented here provide evidence that these two distinct fimbriae are under the control of a two-component regulatory system: FimS/FimR. Expression of major fimbriae (FimA) is extremely low in the *fimR* mutant, and minor fimbriae production in this mutant is inhibited by least 50%. Therefore, it is proposed that FimR can be an attractive target for inhibition of *P. gingivalis* colonization.

## Statement

Re-use of this article is permitted in accordance with the Creative Commons Deed, Attribution 2.5, which does not permit commercial exploitation.
